# Polyacetylene: Myth and Reality

**DOI:** 10.3390/ma11020242

**Published:** 2018-02-06

**Authors:** Bruce S. Hudson

**Affiliations:** Department of Chemistry, Syracuse University, Syracuse, NY 13244-4100, USA; bshudson@syr.edu; Tel.: +1-315-443-5805

**Keywords:** polyacetylene, double-minimum potential, Peierls barrier, zero-point level, cross-linking

## Abstract

Polyacetylene, the simplest and oldest of potentially conducting polymers, has never been made in a form that permits rigorous determination of its structure. *Trans* polyacetylene in its fully extended form will have a potential energy surface with two equivalent minima. It has been assumed that this results in bond length alternation. It is, rather, very likely that the zero-point energy is above the Peierls barrier. The experimental studies that purport to show bond alternation are reviewed and shown to be compromised by serious experimental inconsistencies or by the presence, for which there is considerable evidence, of finite chain polyenes. In this view, addition of dopants results in conductivity by facilitation of charge transport between finite polyenes. The double minimum potential that necessarily occurs for polyacetylene, if viewed as the result of elongation of finite chains, originates from admixture of the 1^1^A_g_ ground electronic state with the 2^1^A_g_ excited electronic singlet state. This excitation is diradical (two electron) in character. The polyacetylene limit is an equal admixture of these two ^1^A_g_ states making theory intractable for long chains. A method is outlined for preparation of high molecular weight polyacetylene with fully extended chains that are prevented from reacting with neighboring chains.

## 1. Introduction/Background History

Polyacetylene is selected for review because of its relative simplicity; the small periodic repeat permits polyacetylene to be treated by sophisticated computational methods. The path from bond-alternate potential minimum to symmetric bond-equivalent maximum is along a single normal mode (the Peierls mode). The vibrational spectrum of polyacetylene is relatively simple. In particular, the Raman spectrum is quite sparse and the oligopolyenes that lead to polyacetylene show characteristic frequency shifts and relative intensity changes which increase in chain length. On the experimental side, however, polyacetylene is entirely insoluble, reactive with itself, and has not been obtained in crystalline form that yields to single crystal diffraction, a feature that it shares with most polymers. Another reason for this review is that there have been several recent experimental and theoretical studies of polyacetylene and finite chain oligopolyenes of known length that are relevant to studies of the structure of polyacetylene. Furthermore, the evidence for the existence of bond alternation in polyacetylene has never been critically reviewed. This exercise shows that the fully extended chain with an all *trans* geometry has never been made. We outline a new method for the preparation of polyacetylene with these properties.

The first publication on the electrical conductivity of doped polyacetylene in 1977 [1], and in a recent 2017 review [2], it is stated as known that *trans*-polyacetylene exhibits bond alternation as a consequence of Peierls instability. It should be noted that Peierls instability refers to a negative curvature in the potential energy for a one-dimensional lattice. The resulting “dimerization”, if it occurs, would mean that the periodic repeat for the electron exchange integral is two CH units, and thus the material has filled bonding and empty antibonding π-orbital bands. Thus, *trans* polyacetylene in its fully extended periodic form would be a semiconductor if it has bond alternation and a conductor if it does not. A close reading of the literature suggests an alternative interpretation of the argument: “since polyacetylene is not a conductor, it must be a semiconductor, and thus it must exhibit bond alternation”. The alternative to this argument is that “polyacetylene” is really a mixture of finite chains and cross-linked polymer segments. 

In this first of many papers [1], it was noted that bond alternation can also be inferred in long linear conjugated polyenes because the allowed optical “band gap” transition converges to a constant value with increase in conjugation length. An alternative view based on strong electron correlation for this limit that does not require bond alternation [3] in order to exhibit a finite limiting electronic excitation energy was mentioned in this initial work [1]. It is argued below that this alternative Mott semiconductor (Hubbard Hamiltonian) picture is substantially correct for very long chains.

Bond alternation is the key ingredient in the interpretation of the observations for polyacetylene in terms of a semiconductor band gap and “doping” enhancement of electrical conduction. In the recent review [2] it is stated that this Peierls barrier is very high on the basis that polyacetylene cannot be made to have equal bond lengths at any reasonable temperature. This proposed double well potential is rarely shown with a quantitative energy scale. The Peierls effect in the case of a π-electron system with a stiff σ-framework might lead to a negligibly small barrier. It is only in classical mechanics that atomic positions are at the bottom of the potential. 

The earliest reasonable estimation of the “dimerization energy” (Peierls barrier) is a 1983 work [4], where Hartree Fock and small basis set MP2 (Moller-Plesset second order) methods were used. The MP2 value for the difference in energy at the symmetry point and either minima of the potential is 2 kJ/mol (700 cm^−1^) [4]. A more extensive 1997 treatment [5] used multiple linear conjugated polyenes of increasing length, for which optimized structures were compared with equal bond structures or with structures that had one of several variations of bond alternation with optimized bond lengths at the molecular ends changing to equal bonds in the middle of the chain. The resulting dimerization energy extrapolated to 1/N = 0 at the MP2/6-31G* level was 0.4 ± 0.1 kJ/mol depending on the method of structure variation used. The low end of this range is 0.3 kJ/mol or 105 cm^−1^. The point that has never before been considered in this context is that the harmonic vibrational motion at the bond alternation geometry has a frequency known since at least 1958 to be in the 1500–1600 cm^−1^ range corresponding to a double bond stretching motion [6,7,8]. The harmonic zero-point energy is higher than the estimated barrier height.

We have shown, as discussed below, that while polyacetylene must have a potential energy surface with two equivalent minima, it cannot exhibit bond alternation, i.e., a periodic alternation between short and long bonds. The band structure argument that leads to this conclusion, leads conversely to the conclusion that if polyacetylene did not have bond alternation, it would be metallic. It would appear, however, that polyacetylene is not metallic unless it is “doped” with electron donors or acceptors. The terminology is from elemental semi-conductors where the doping is elemental. Here the “dopant” is usually molecular. Thus, the experimental evidence appears to differ from a conclusion in regard to ground state structure of polyacetylene that seems elementary. The alternative point of view investigated in this paper is that this conflict between our conclusions regarding the necessary lack of bond alternation of polyacetylene and experimental results for so-called “polyacetylene” is that what is called “polyacetyene” is not in fact polyacetylene, but is, rather, a mixture of finite chain polyenes of various lengths. The addition of dopants permits electron transport from chain to chain. These oligopolyenes do exhibit bond alternation and give rise in spectroscopic studies this property but this is due to end effects.

The term “finite polyene” here means that the molecule exhibits bond alternation with the terminal carbon–carbon bond length being shorter than the average of the values near the center of the molecule by an amount of 0.003 Å or more; barely measurable but otherwise arbitrary. We do not know and cannot easily compute how long a polyene needs to be such that it has two minima in its bond alternation potential and further how long it must be to have two equal energy minima. Whatever that is, it defines “approaching infinite”. Minima of exact energy equality energy define “infinity” for the chain length.

To reiterate, our conclusion is not that polyacetylene has a single minimum potential energy for the bond alternation atomic displacement mode, but rather that the zero-point level is above the barrier that separates two minima. It is the probability distribution of the zero-point barrier that determines the structure, not the minima of the potential. Both minima are equally populated in the zero-point probability distribution. Simulations with reasonable parameters predict, in fact, that the maximum of the probability distribution is at the symmetry point where the potential is a maximum.

When referring to studies of the preparations, we use the notation “polyacetylene”. We restrict the terms *cis-* or *trans-*polyacetylene to hypothetical infinite chains in their fully extended conformation. We are primarily concerned here with *trans*-polyacetylene. An infinite transationally symmetric chain is the starting point for the standard treatment of polymer vibrations in general as developed by Born and von Karman. As in all periodic problems, the description of the nuclear motion is the product of a local function times a function with translational symmetry along the chain propagation direction. It is the vibrational levels of the periodic repeat unit that count in establishing the zero-point level energy and the “optical” vibrational excitations in infrared and Raman spectra.

The relevant internal degree of freedom in this case is the bond alternation or Peierls distortion mode, a mixture of double bond expansion and single bond contraction. The potential energy in which the nuclei move for this degree of freedom necessarily has two minima that are exactly equivalent in energy only for the infinite chain. The equivalence of the minima derives from the fact that the energy difference between the two patterns of bond alternation becomes negligibly small per repeat unit as the chain length becomes very long. This argument makes no statement as to the height of any barrier between the two minima, if there is one.

Assuming that there is a barrier, then the issue of how to treat the nuclear motion arises. In this regard, there are two relevant observations. (1) The harmonic vibrational frequency that corresponds to motion along the Peierls degree of freedom is computed to have a value in the harmonic approximation of ca. 1500 cm^−1^. This corresponds to the strongest Raman active mode at a similar wavenumber. (2) The best estimate of the height of the Peierls barrier via extrapolations discussed above is 100–300 cm^−1^ [5]. The harmonic zero point level is thus 2–7 times larger than this barrier height. Use of the harmonic approximation is clearly not justified.

## 2. Summary of This Review

We first review in Section 3 the double minimum problem in general, for two molecular cases, and then in Section 4 the specific case of polyacetylene. This is followed in Section 5 by a survey of experimental observations on polyacetylene in the literature that are relevant to bond alternation. It is found that X-ray diffraction, solid state NMR (Nuclear Magnetic Resonance), and polarized IR studies are compromised by ambiguities internal to the studies or to the presence of the finite chains, or both. The electronic spectroscopy of finite conjugated polyenes is then discussed in Section 6. The conclusion of these spectroscopic studies is that a low-lying doubly excited “diradical” state with the same symmetry as the ground electronic state is the lowest energy electronic excitation. This conclusion for the best studied case of octatetraene has recently received theoretical treatment, whose results are in excellent agreement with experiment. Admixture of this 2^1^A_g_ excited state with the ground 1^1^A_g_ state is the origin of the double minimum barrier for polyacetylene in its ground electronic state. It is also the basis of the difficulty in dealing theoretically with the ground state of polyacetylene with current periodic quantum chemical computational methods, since it requires inclusion of at least all doubly excited configurations at a non-perturbative level. This is followed by a brief review in Section 7 of the experimental electronic and vibrational Raman spectra of finite linear polyenes facilitated by the recent availability of such materials in homologous series. In Section 8, it is shown how these Raman spectra are relevant to our ongoing experimental Raman and vibrational inelastic neutron scattering studies of a molecular crystal for which photochemical elimination polymerization has been demonstrated to occur that leads to polyacetylene constrained to be fully extended in parallel channels formed by an inert lattice that also prevents cross-linking reactions. The iodine atoms that are photochemically cleaved are able to leave the host crystal as iodine vapor. In Section 9, the salient features gleaned from the literature are reviewed and an outlook is presented.

## 3. Double Minimum Potential Vibrational Energy Levels: Ammonia and [18]-Annulene

**Figure 1:**
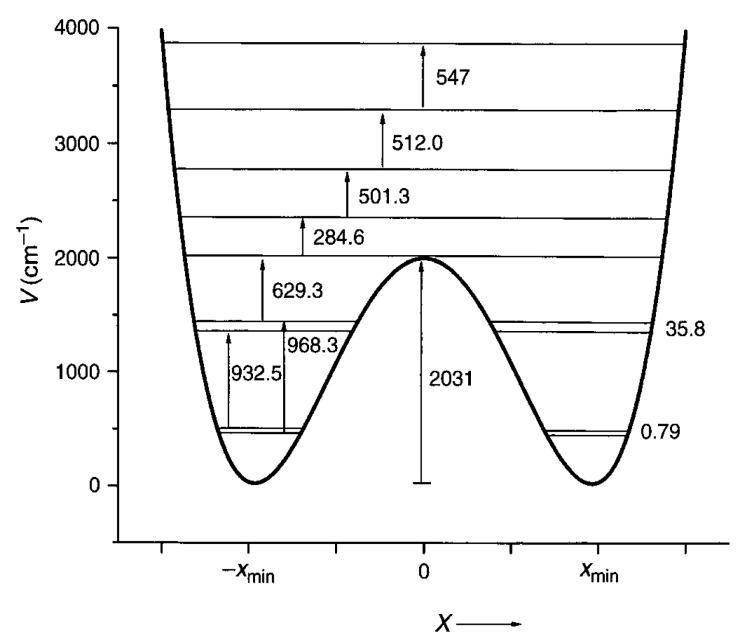
Umbrella mode potential for NH_3_ with transition and level splittings indicated [9,10].

The mathematical technology for determination of the vibrational energy levels of arbitrary one-dimensional potential is now straightforward. These methods were developed to treat numerous molecular potentials [9,10,11,12,13,14,15] that have two equivalent minima. The most famous of these is ammonia, where the tunneling splitting is ca. 0.8 cm^−1^. A potential that fits the precise vibrational data is shown in Figure 1 [9,10,11,12,13]. A potential that has the form V(x) = C_2_x^2^ + C_4_x^4^ with C_2_ = −9000 cm^−1^ A^−2^ and C_4_ = 10,000 cm^−1^ A^−4^ and a reduced mass of 1.008 amu has a tunneling splitting of 0.45 cm^−1^ (vs. 0.79 cm^−1^ of Figure 1). The 0 to 1 transitions of 932.5 and 968.3 cm^−1^ are computed to be at 940.3 and 969.8 cm^−1^. The barrier height of 2031 cm^−1^ is 2025 cm^−1^ in this simple treatment using the efficient FGH (Fourier Grid Hamiltonian) method [14,15]. The reduced mass for ammonia varies along the out-of-plane umbrella coordinate. For the equilibrium pyramidal geometry, the value is 1.18 amu, while at the trigonal D_3h_ maximum it is 1.20 amu. This increase relative to the mass of H reflects the small geometry-dependent contribution of the N atom to the inversion normal mode. The zero-point level tunneling splitting of ammonia corresponds to an inversion time for the pyramidal superposition state of about 11 ps. This follows from the tunneling splitting 0.45 cm^−1^ for NH_3_ in the simplest model treatment. This same model gives 792 ps for the tunneling splitting for ND_3_. 

**Figure 2:**
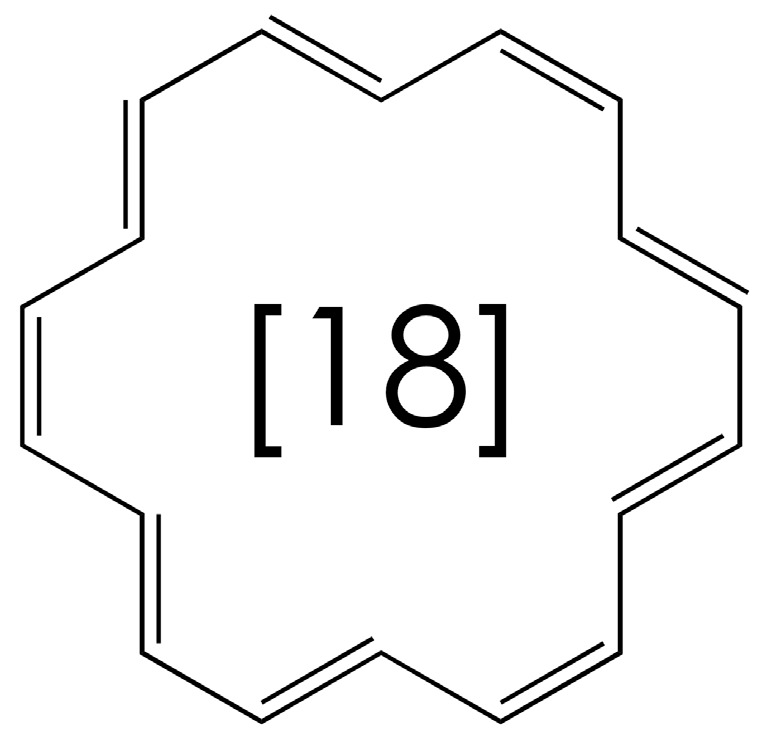
One of the D_3h_ Kekule structures of [18]-annulene.

**Figure 3:**
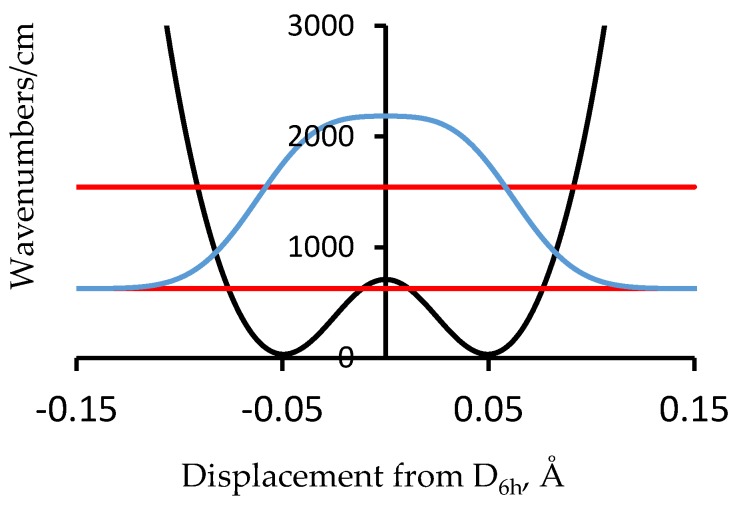
Computed potential energy as a function of displacement from 6-fold symmetry for [18]-annulene (black line) showing the two lowest vibrational energy levels (red) and the probability distribution for the ground state (blue) [17].

Another molecular example of more relevance to polyacetylene is [18]-annulene, Figure 2 [16,17,18]. This simple cyclic C_18_H_18_ compound is the 4*n* + 2 analog of benzene (*n* = 1) with *n* = 4, and is thus expected to be aromatic. To make a complicated story short, this conclusion is consistent with the observation of six-fold equivalent bonds in the X-ray diffraction structure but not with the computed NMR spectrum (for which the inside and outside protons are not shifted in opposite directions by the same amount as is the case for the D_6h_ symmetry). It has been proposed that [18]-annulene has a D_3h_ bond-alternate structure. A method of computation is found that results in a D_3h_ bond-alternate structure that results in agreement with the NMR spectrum [16]. This proposed geometry is either one of the structures corresponding to the minima of the potential in Figure 3. The zero-point level and probability distribution are shown. This proposed geometry is either one of the structures corresponding to the minima of the potential in Figure 3. The zero-point level and probability distribution are shown. A vibrational normal mode analysis at the symmetry point maximum and also at the minima gives in each case a reduced mass of 9.315 amu. The proton NMR spectrum computed for 200 points along bond order displacement coordinate weighted by the probability of Figure 3 gives a value in reasonable agreement with experiment. Other details of this density functional theory (DFT) and FGH treatment for [18]-annulene are in [17]. A classical MD (Molecular Dynamics) treatment for NMR averaging that includes this case is in [18]. An important factor for this case is that one of the normal modes of this molecule converts the structure from the maximum of the potential to either one of the minima and back. This example provides a demonstration that zero-point heavy atom averaging is expected in such cases because of the very stiff nature of the bonds prohibits localization into one of the minimum energy wells. The general point to keep in mind is that even with heavy atom motion, it is impossible to localize a carbon-based structure into a localized bond-alternate structure for a period of time that is significant on an experimental time scale. Benzene is the obvious example.

## 4. Double Minimum Potential Vibrational Energy Levels: Polyacetylene

For cases like ammonia, where the double minimum potential represents displacement of the three H atoms out of the molecular plane, and this case of a cyclic hydrocarbon, the potential must necessarily contain only even terms. The potential energy variation for polyacetylene must also necessarily be symmetric due to translational symmetry.

**Figure 4:**
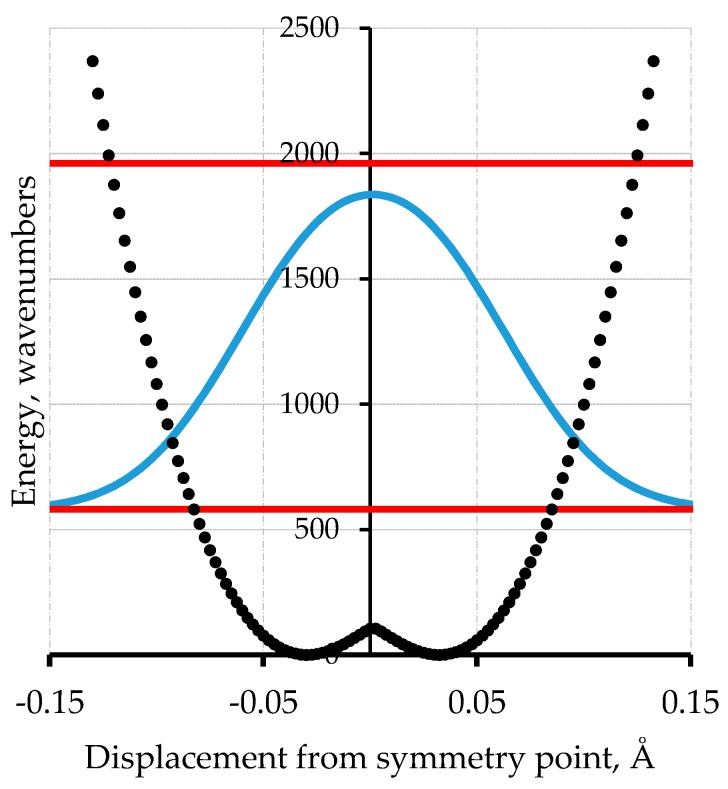
Computed potential energy of polyacetylene using periodic boundary conditions-density functional theory (PBC-DFT) with B3LYP 6-311G(2d,2p) at 240 points (black points) along one displacement direction with subsequent generation of the symmetric potential shown as blue dotted trace [19]. The horizontal red lines are the two lowest energy levels; the light blue line is the probability distribution.

**Figure 5:**
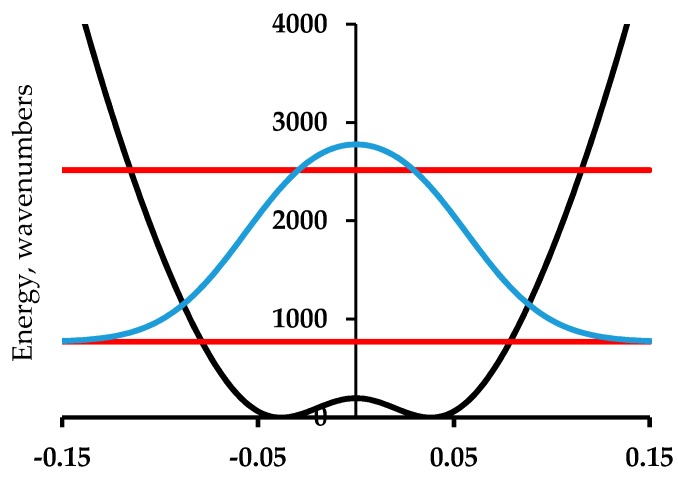
An analytical model potential energy (black curve) for the Peierls bond alternation mode of polyacetylene. The function is a harmonic oscillator plus a Gaussian barrier [19]. The lines are as in Figure 4.

For the case of polyacetylene [19], for which periodic boundary conditions [20] apply, we have followed two independent paths of enquiry in Figure 4 and Figure 5. In Figure 4, we compute the energy of the –CH–CH– periodic repeat using B3LYP/6-311G(2d,2p) with periodic boundary conditions evaluated at 240 points along the potential in one direction. This is then symmetrized by reflection. The barrier height computed by this DFT method is 110 cm^−1^.

Figure 5 shows the results of an empirical treatment of polyacetylene. The barrier height for polyacetylene has been established by the extrapolation procedure of Guo and Paldus [5] to be less than 200 cm^−1^. In their treatment, a series of computational methods are applied to three structures for each member in a series of a finite polyene chains with an even number of carbon atoms. The structures are (1) the optimized bond-alternate structure, (2) the equal bond length (barrier) structure, and (3) the bond order reversed structure corresponding to the other minimum in the infinite chain case. These energies values relative to the optimized structure are plotted against 1/N, where N is the number of C=C bonds. The values of this energy difference for the bond reversed and bond optimized cases must, of course, extrapolate to zero as 1/N goes to zero. The plot for the barrier height when extrapolated linearly gives a finite value of about 100 cm^−1^. Figure 5 uses a barrier of 200 cm^−1^. This value comes from the observation that for chain lengths that are sufficiently long, the computed values before extrapolation are below that value, so this value is an upper limit. The harmonic force constant for the model of Figure 5 is chosen to match the value of the force constant for C–C single bonds based on harmonic normal mode analyses for simple molecules like ethane. This is the lowest reasonable value. Higher values of this force constant parameter will result in a higher zero-point energy. The reduced mass for both cases is 4.33 amu. This is derived from a Gaussian computation for finite polyenes which uses the Wilson, Decius & Cross prescription [21]. This value depends on the C–C–C bond 120° C–C–C angle but is not crucially dependent on this value.

Calculations of the vibrational frequencies of long linear polyenes using MP2 wavefunction methods has been used to compute the force constants needed for harmonic treatment of polyacetylene in the periodic limit. It is found from the resulting vibrational eigenvalues that the vibrational motion that gives rise to the strongest feature in the Raman spectrum is the bond alternation or Peierls motion mode. Figure 4 and Figure 5 show the computed first excited vibrational mode for these potentials are at 1379 cm^−1^ for the DFT computed potential and at 1460 cm^−1^ for the variable parameter treatment. At the time of this work, it was thought that the strongest Raman active mode of what was thought to be polyacetylene was at 1459 cm^−1^. The 1460 cm^−1^ value was chosen as a target value in adjusting the barrier width of this analytic empirical model of Figure 5.

If this modelling procedure is followed with larger values of the barrier height including readjustment of the harmonic force constant so that the same value of the vibrational frequency is obtained, then with a barrier height of 2000 cm^−1^ the ground state zero-point level has a double maximum. Because of the symmetry, the probability of being in one well is the same as being in the other in this and every other state. If the barrier height is raised to 20,000 cm^−1^, then the energy levels occur in pairs with a splitting for the lowest level of 20 cm^−1^ corresponding to femtosecond time scale tunneling. Bond-order alternation states will be exceedingly ephemeral.

## 5. Review of Experimental Observations on Polyacetylene with Emphasis on Bond Alternation

In the literature, especially in theoretical publications, bond alternation is often used to mean that there are two equivalent minima in the potential energy function. It is our argument above that this cannot give rise to populated bond-alternate structures with a periodic difference in bond lengths. If the zero-point energy is considered, then both structures are equally populated and, for what is considered to be at least a reasonable approximation to reality, the most probable geometry is at the symmetry point were the two bond lengths are equal. It might be argued that bond alternation in polyacetylene is known to occur on experimental grounds. This is, however, not the case when the experimental studies are viewed critically taking into account the likely presence of finite chains in the sample. 

This section on experimental observations is divided into (1) X-ray diffraction, (2) infrared dichroism, (3) solid state NMR spectroscopy, (4) resonance Raman spectroscopy, and (5) a cautionary note on doping.

### 5.1. X-ray Diffraction

We begin with X-ray diffraction studies of polyacetylene. The initial 1982 work in this field [22] is discussed in a 1984 monograph [23], re-evaluated in a 1992 experimental study with new X-ray data [24], and then discussed in a 2010 computational paper [25]. Beginning with [22], it is noted that there are two possible monoclinic structures with two chains per unit cell P2_1_/a and P2_1_/n corresponding to the case of in-phase bond alternation or out-of-phase, respectively. For the former P2_1_/a in-phase case, the (001) reflection is expected to be strong; for P2_1_/n, it is forbidden. In this experiment [22], the (001) reflection is observed very weakly. The information in [22] as to bond alternation stems from an analysis of the shape of this (001) reflection with a two-parameter least squares fit of the alteration parameter u_0_ and the monoclinic angle β to data with an intensity of unspecified physical origin, that depends on sample preparation, that clearly consists of more than one reflection and for which the statistics of the fit are not reported. The data presented in [22] are critically analyzed in [23] and in particular, the two parameter fit was repeated. No distinct minimum was observed for the least squared fit. The newer 1992 experimental study with new data of [24] took a global look at the full data set. It was decisively determined that the structure is P2_1_/n with expectation of a forbidden (001) but again, observed weakly. The authors note, “A possible explanation of the (001) intensity might be the coexistence of a small, variable fraction of bulk P21/a second phase. However, in our sample the (00L) intensities imply 20% P2_1_/a while the ratio of I(021)/I(011) gives an upper limit of only 4%. This inconsistency between on-axis and off-axis measures rule out the phase separation argument.” They continue, “An alternative explanation is that local defects are responsible for the (001) intensity. For example, since the energy difference between the two structures is small, one might envision defects such as short chain segments which correlate in-phase with the surrounding long chains. Such defects would be a natural consequence of small molecular weight fragments, and could easily be quenched in from the polymerization. They would contribute to the (001) but not to general (HKL) intensities as is observed.” Or they could be low molecular weight interstitial oligopolyenes. In the analysis of this data [24], the bond alternation parameter was not adjusted to fit the data. Instead, a value determined by NMR was used. This removes this X-ray study from having an impact on the bond-alternation issue. The validity of this NMR determination is discussed below.

### 5.2. Infrared Dichroism

Infrared (IR) absorption dichroism in the spectral region of the CH stretch [26] has been used to argue that polyacetylene exhibits bond-alternation. If polyacetylene has equal bond lengths for sequential bonds, then the 3013 cm^−1^ C–H stretch absorption will be, by symmetry, perpendicular to the chain (and thus perpendicular to the stretch direction for a stretched film). The absorption will be zero when the electric field lies along the orientation axis. It is found that this is not the case. The out-of-plane bending mode exhibits significant dichroism in the direction expected, indicating that the chain is well oriented. It was concluded that the local symmetry is C_s_, not C_2v_, permitting a dipole derivative with a component along the chain axis. However, a DFT calculation for the finite polyene chain C_60_H_62_ gives the dipole derivative for the IR-active CH mode at an angle of only about 25 degrees with the extended chain. This is presumably due to the very large axial polarizability. This axial component, however, vanishes in the postulated symmetric structure. It is expected that longer oligopolyene chains will have a larger axial/transverse ratio, and it is quite possible that the long-chain part of the distribution may dominate the IR spectrum, even if they are not the predominant species in the sample. The interpretation of this experiment depends on whether the signal is dominated by finite chains in the sample or by polyacetylene. This depends on both the amounts of these components and their relative intensities.

### 5.3. NMR Spectroscopy 

The simplest indication of the nature of polyacetylene at the molecular level is the determination by CP MAS (Cross Polarization Magic Angle Spinning) ^13^C NMR that ≈5% of the carbon atoms have sp^3^ hybridization instead of the predominant sp^2^ hybridization. The sp3 features “can probably be ascribed to chain terminations, cross-links, or hydrogenated double bonds” [27]. This corresponds to one atom in 20. If we ascribe the putative sp^3^ carbons to cyclobutane ring formation then this corresponds to two cyclobutane defects in 200 carbons or 50 C=C double bonds from one to the next in two chains connected by four sp^3^ carbons in cyclobutane rings at each end. This is, of course, only the average structure in a distribution.

Another NMR experiment aimed directly at detection of bond alternation is the determination of the ^13^C–^13^C dipolar coupling constant of polyacetylene prepared with low levels of acetylene–^13^C_2_ [28,29]. In this case, the observation of two coupling constants for *trans*-polyacetylene indicates two distinct bond lengths. This work has led to the prevailing bond alternation value and the individual C–C bond lengths for polyacetylene. For example, [30] compares a computed value to the bond lengths in [28]. There are, however, a few areas of concern in this work. The *cis*-polyacetylene isomer shows, as expected, only one coupling constant corresponding to a double bond length, 1.37 Å. Converting the sample of *cis*-polyacetyene to *trans*-polyacetylene is done by heating in vacuum at 160 °C for one hour. The resulting solid state nutation NMR spectra at 77 K show two coupling constants corresponding to 1.36 and 1.44 Å bond lengths. It is noted [28] that “The generation of approximately equal populations of singly and doubly bonded labeled carbon pairs in *trans*-(CH)_x_ starting with only doubly bonded pairs in *cis*-(CH)_x_ is intriguing.” No explanation is given for this observation. The only obvious explanation is that half of the ^13^C=^13^C double bonds react with nearby predominantly ^12^C=^12^C double bonds to make ^13^C–^13^C single bonds from the original ^13^C=^13^C double bonds in cyclobutane rings. This is the kind of unambiguous experiment that is not done often enough. Presumably this also happens with half of the ^12^C=^12^C bonds. The result is best called *poly(“ladderane”).* Performing a CP-MAS ^13^C determination of random ^13^C labeled polyacetylene treated in the same fashion. Both before and after thermal conversion to the *trans* form would be interesting.

The authors further investigated the temperature dependence of the ^13^C–^13^C splitting up to 300 K and did not see an expected coalescence of the features from defect migration along long chains. They speculated that the signals that they were observing came from chains that did not contain mobile defects necessary for thermal averaging, i.e., locked-in bond alternation due to cross-linking. In another work [29], it was concluded that “nuclear spin-lattice relaxation rates for ^1^H and ^13^C in polyacetylene cannot be adequately explained in terms of either nuclear spin diffusion to a static paramagnetic defect or rapid one-dimensional diffusion of the defect itself. A model in which only a small fraction of the molecular chains contain defects was proposed. Nuclei on these chains are rapidly relaxed, whereas the remainder achieve equilibrium by nuclear spin diffusion. The dependence of the measured relaxation rates upon frequency and isotopic concentration agreed with the predictions of the model.” As indicated by the above, an alternative hypothesis for this signal is that it comes from finite polyene chains.

### 5.4. Resonance Raman Spectroscopy

Resonance Raman studies of “polyacetylene” [31,32,33,34,35,36,37,38,39,40,41,42,43,44,45,46] make use of the fact that Raman scattering under resonance conditions has a greatly enhanced contribution from species that have electronic excitation resonances near the Raman excitation frequency. If the sample is homogeneous, then the intensity of vibrational features in the resonance Raman spectrum will all increase or decrease together as the excitation frequency is changed. If, however, the sample is heterogeneous, with various components having different electronic excitation behavior and vibrational spectra, changing the excitation frequency will cause some vibrational features to increase and other decrease in intensity. In the case of “polyacetylene” containing multiple oligoene components, the electronic excitation spectrum moves to lower energy, and the strongly enhanced vibrational mode moves to lower frequency, as the chain length is increased. It is observed as expected that Raman excitation at longer wavelength results in lower frequency Raman active modes. The conclusion of most of these studies is that chain length heterogeneity is the most likely explanation for the experimental observations. Simple calculations reproduce the data, and alternative models were eliminated [43].

Finite linear conjugated polyenes have very strong Raman scattering. This is especially true when the Raman excitation is close to the strong electronic absorption bands of polyenes. The reason for the very strong Raman scattering of polyenes is that linear polyenes have a very strongly allowed electronic excitation that involves an appreciable geometry change in the excited state relative to the ground state. This geometry change is primarily along one normal mode of motion in the ground state near 1500 cm^−1^ with a smaller contribution from another mode around 1100 cm^−1^. The details of this are discussed in Section 6 below. Here, we concentrate on “polyacetylene” in the context of its anticipated Raman scattering. This issue was noted in [44] in the context of periodic solids and in a way relevant to this review in [45,46]. In these publications, it is noted that the Condon mechanism for Raman scattering (also called A-term scattering) due to geometry changes in excited electronic states vanishes for periodic solids and for polyacetylene as we have defined it. This is because the one-electron excitation involved does not result in a significant geometry change for a system of effectively infinite size. The most argumentative statement of the issue is in [46], in which it is claimed that (a) since “polyacetylene” has a well-known Raman spectrum, it must be the case that something is missing that is beyond the Condon scattering mechanism; (b) a vibronic activity term is added (called B-term scattering), which (c) explains (“solving polyacetylene”) by addition of the next term albeit with an unknown magnitude, and (d) that this refinement also removes the need to consider polyacetylene as a heterogeneous mixture of finite chains [31,32,33,34,35,36,37,38,39,40,41,42]. Specifically, quoting from [46], “In ref. 24 (of [46]), three samples of nearly monodisperse polyacetylene with lengths of about 200, 400, and 3800 unit cells were synthesized and their Raman spectra were obtained. The sidebands remained, and many of the earlier “polydisperse” explanations for the line shape quickly evaporated.” The relevant ref. [24] is here ref. [35].

The authors of [46] have misrepresented [35] by claiming that the samples involved have very long conjugation length and ignoring the fact that in [35] itself these “monodisperse” samples are investigated as to the polydispersity of the conjugation lengths. Quoting from the abstract of [35] “After thermal isomerization, theoretical analysis of the Resonance Raman spectra using the Brivio, Mulazzi model indicate the ratio of long trans conjugated segments (N ≥ 30) to short trans conjugated segments (N ≤ 30) is significantly larger for 100,000 Dalton (3800 unit) polymer.” It is the contention of this author that it is the N = 15–30 and perhaps a bit longer double bond species that have Raman spectra from the Condon mechanism, and that there is no evidence that anything that might be defined as polyacetylene makes any contribution to the Raman spectrum.

### 5.5. A Cautionary Note on Doping

Our argument, outlined above, is that it is not possible that the nuclear probability distribution will exhibit bond alternation in any experiment. The reported bond alternation in “polyacetylene” is evidence for the presence of finite chains. These may dominate the signals even if they are not the major component of the sample itself. The “semiconductor” properties attributed to “polyacetylene” are, in this interpretation, due to the lack of extended conducting chains. The observed effect of “doping” on conductivity, interpreted in terms of band theory, may rather be due to enhancement of conduction via electron transfer between finite chains rather than population of a conduction band, as assumed in the conventional model. In fact, iodine “doping” of polymers such as polybutadiene that do not contain any conjugated double bonds results in significant conductivity [47,48]. This is attributed [47] to the presence of ionic iodine chains such as I_3_^−^ and I_5_^−^ in the material. In [47], the conduction is claimed to be electronic.

## 6. Electronic Spectroscopy of Finite Linear Conjugated Polyenes

Interest in the electronic spectroscopy of linear conjugated polyenes began in the early days of the development of quantum mechanics. At that point, dealing with polyatomic molecules was based on qualitative molecular orbital and valence bond descriptions. Early treatments of butadiene with valence bond methods concluded that the lowest energy excitation would be a diradical with the same A_g_ symmetry in C_2h_ as the ground state. The observation of a strongly allowed electronic excitation contradicted this view. For linear conjugated polyenes with an even number of carbons in the C_2h_ point group, the symmetries of the non-degenerate molecular orbitals alternate in symmetry with increasing energy with a behavior similar to that of a particle in a box. Because of this, the HOMO-to-LUMO excitation is thus necessarily from an A_g_ ground state to an excited state of B_u_ electronic symmetry. This seemed to be in agreement with experiment in respect to a low-energy, strongly allowed transition that increased in intensity and decreased in energy as the polyene chain increased in length. There were, however, several aspects of linear conjugated polyene electronic spectroscopy and, especially, of the corresponding photophysics that attracted our attention that all was not right. The main things that we found of interest was the observation [49,50] that the intrinsic fluorescence (or radiative) lifetime of linear polyenes is much longer than expected on the basis of the integration of the absorption spectrum. The other items of interest were the series of spectroscopic papers on linear polyenes by Hauser and co-workers [51,52,53,54,55,56] and the discussion of that work by Mulliken [57]. In this discussion, Mulliken said:“A puzzling phenomenon reported by Kuhn, Hausser, and co-workers in their comparative study of absorption and fluorescence in polyene derivatives is the existence of a gap between the longest wave-length absorption band in the vibrational structure of N to V_1_ and the shortest wave-length band the corresponding V_1_ to N fluorescence spectrum. It is worth noting, however, that there seems to be still a small amount of absorption and emission at the middle of the gap. The width of the gap increases with increase in the number of conjugated double bonds. The absorption and fluorescence spectra are roughly mirror images of each other on a frequency scale. There seems to be no reason, especially in view of our theoretical analysis, to doubt that the fluorescence spectrum really is V_1_ to N. According to the theory, no other excited singlet level should be below V_1_.” This is followed by an attempt to explain the observed spectral pattern in terms of an in-plane bending deformation of large amplitude. A similar explanation was posed for the long intrinsic lifetime [49,50]. 

**Figure 6:**
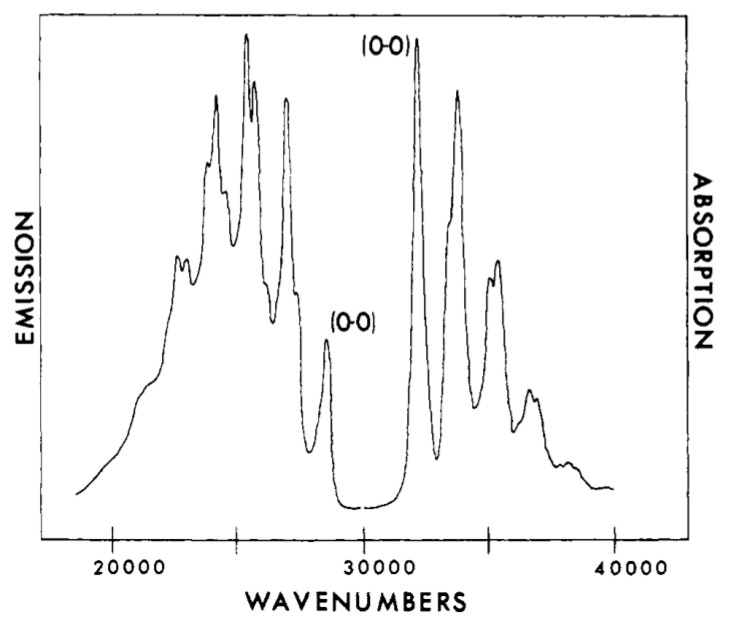
Fluorescence and absorption spectra of all *trans*-1,3,5,7-octatetraene in 3-methylpentane at 77K. Left, fluorescence on an arbitrary emission scale; right, absorption on an arbitrary absorbance scale [58].

The partially resolved vibronic spectra (Figure 6) of absorption and fluorescence of 1,3,5,7–octatetraene at 77 K have a gap between what appears to be the first absorption feature (the 0-0) near 32,000 cm^−1^ and the first fluorescence feature near 29,000 cm^−1^. This is not anticipated if there is only one low-energy excited electronic state. On the other hand, if there is another low-lying state, this absorption should begin where the fluorescence begins near 29,000 cm^−1^.

**Figure 7:**
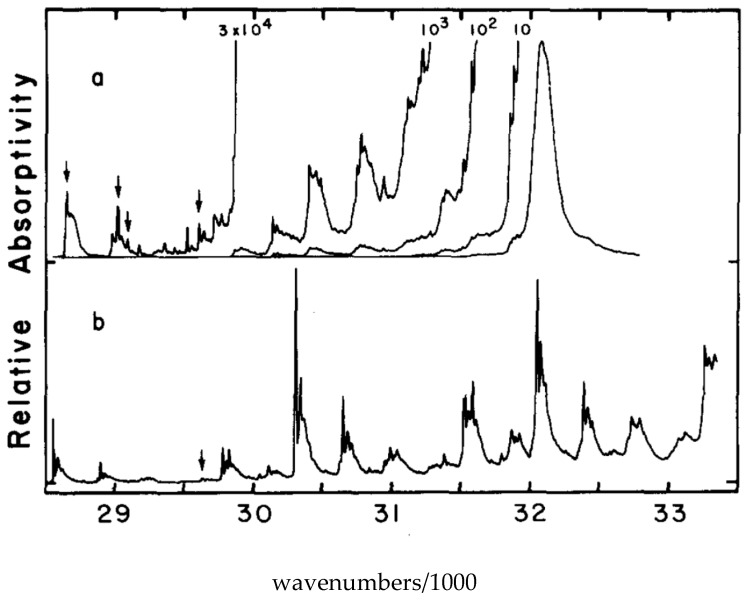
(**a**) The one-photon fluorescence excitation spectrum of octatetraene in n-octane matrix at 4.2 K. The arrows mark the vibronically induced transitions to the forbidden 2^1^A_g_ excited state. The intense broad feature at 32,200 cm^−1^ is the vibration-less origin of the allowed electronic transition to the 1^1^B_u_ excited state; (**b**) Two-photon fluorescence excitation spectrum of the same sample. All of the features are due to transitions to the 2^1^A_g_ excited state. [59,67,68].

**Figure 8:**
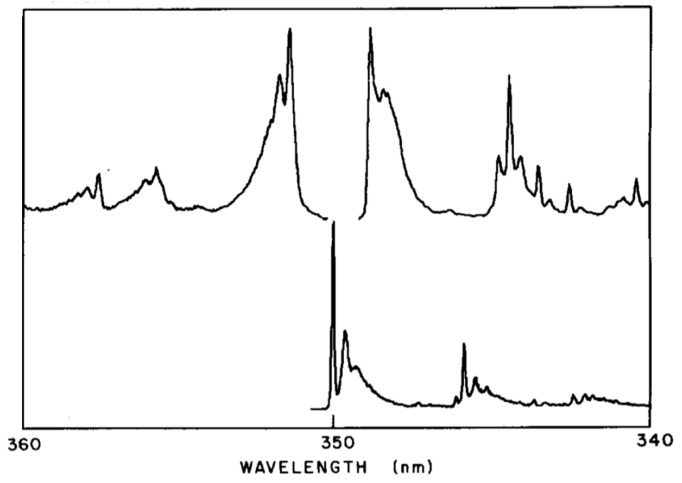
The upper trace left is the beginning of the fluorescence spectrum; the upper trace right is the beginning of the one-photon fluorescence excitation spectrum; the lower trace is the beginning of the two-photon fluorescence excitation spectrum. The two traces on the right are the same as the extreme left of Figure 7. [59,67,68].

In order to observe the hidden spectral features, it is necessary to use a solvent that at low temperature provides the same environment for all dissolved solute octatetraene molecules. This was found to be the case for n-octane, where apparently a centrosymmetric mixed crystal is formed. The spectra shown in Figure 7 and Figure 8 are for polycrystalline matrices of n-octane with a very low concentration of octatetraene. The major features in these spectra are (a) the vibration-less origin (0,0) transition of the 1^1^A_g_ to 1^1^B_u_ electronic transition near 310 nm (32200 cm^−1^ in Figure 7), (b) the first absorption transition to the new low-energy, low-intensity transition near 348 nm (Figure 8) (28,730 cm^−1^ in Figure 7), and (c) the first emission transition from the as of yet unknown excited electronic state to the ground electronic state of 1^1^A_g_ symmetry near 352 nm in Figure 8. The line shapes reflect phonon side-band structure. 

Figure 8 is an expanded view of the origin region combining with addition in the upper trace the first one-photon feature of Figure 7. The two central features of the upper trace are “false origins” due to modes of b_u_ vibrational symmetry in the upper or the ground state. The lower trace is the two-photon excitation spectrum showing the true 0-0 at 350 nm plus phonon side bands and the lowest molecular a_g_ mode an in-plane bending vibration. These spectra present the classic pattern of Herzberg–Teller vibronic coupling, in which an electronic transition that is forbidden by symmetry is made allowed by a vibronic promoting mode that is non-totally symmetric and thus transiently reduces the molecular symmetry. The absence of the true origin transition in the one-photon excitation spectrum is a result of strict centrosymmetry in the n-octane crystal. The electronic symmetry of the ground electronic state is ^1^A_g_ and so the upper level must also be ^1^A_g_. These are designated 1^1^A_g_ and 2^1^A_g_ respectively with the superscript 1 indicating single states. Use of n-nonane or n-heptane in place of n-octane induces significant intensity in the 0-0 transition due to the necessary loss of symmetry. The vibration-less origin transition is two-photon allowed as is observed. The strongly allowed one-photon transition at about 312 nm is ca. 10^5^ times stronger than the first vibronic feature of the excitation spectrum in the 2^1^A_g_ region. The vibronically active normal modes are expected to be of vibrational b_u_ symmetry because of the proximity of the strong 1^1^B_u_ excitation. The two-photon high resolution spectral feature when compared to the high resolution fluorescence spectrum permits determination of the frequency the promoting mode as 86 cm^−1^, which is an observed mode of this b_u_ symmetry. All of the above were the contribution of Bryan E. Kohler and his students [59,60,61,62,63,64] following the present author’s initial contributions [65,66,67,68]. The above argument as to the presence of a low lying 2^1^A_g_ state in octatetraene is airtight on experimental grounds. The development of the theoretical situation showed very early [69,70,71,72] that the low-lying 2^1^A_g_ state is derived from doubly excited configurations that mix with singly excited configurations of the same symmetry to become the lowest excited state. It is now possible to compute the electronic excitations of octatetraene and get very good agreement with experiment using advanced *ab initio* methods [73].

Analysis of the pattern of the vibrational intensity of the strongly allowed absorption transition of spectra, like Figure 6, for a variety of polyenes shows that the features are due to the ca. 1500 and 1100 cm^−1^ C=C and C–C stretching modes. This is the case for both the transitions to or from the upper 2^1^A_g_ electronic state and the allowed electronic absorption transition to the 1^1^B_u_ state. This means that these excited states differ from the ground electronic 1^1^A_g_ state by displacement along the total symmetric double bond and single bond contraction/expansion vibrational modes. The direction of the displacement is to upper states that have a reversal in their bond alternation pattern. The relative intensity of a vibration in an electronic transition is related to this displacement, which leads to finite overlap between the vibrational modes of the two states involved. These overlap integrals are called Franck–Condon factors. This displacement is the major mechanism that results in the vibrational structure and overall width of electronic spectra and in Raman activity of totally symmetric modes. This is called the A-term or Condon contribution to Raman scattering. This depends on the displacement of the potential energy minimum for the low-energy, strongly allowed electronic excitations. In a more general treatment of Raman scattering, there can also be cases where the mechanism of Raman intensity is due to the fact that some displacements of the atoms result in a change in the intensity of the electric dipole transition moment rather than a change in the energy of the potential energy surface that is required for non-zero Franck–Condon factors. This is especially important in electronic transitions that are between states and have electronic symmetries that cause the electric dipole transition moment to vanish at the equilibrium geometry, as is the case for the 1^1^A_g_ to 2^1^A_g_ electronic transitions of linear polyenes giving rise to the pattern of features shown above.

The relevance of the presence of a low-lying doubly excited 2^1^A_g_ state in finite polyenes to properties of polyacetylene, as pointed out by Torii and Tasumi, is that polyacetylene in its ground electronic state must necessarily be an admixture of structures that have the standard pattern of bond alternation with another structure that has its bond order pattern reversed from that of the optimized structure [74]. The result is a polyacetylene with equal bond lengths. This has been investigated by Torii and Tasumi using the CASSCF (Complete Active Space Self Consistent Field) method. Their results for N = 6, dodecahexaene with an STO-3G basis set give an energy difference per CH group for the optimized geometry and that of the bond-reversed geometry of 2000 cm^−1^. A slight inflection in the potential hints at the evolution toward a double minimum expected for longer chains.

There have been several recent synthetic efforts at preparation of oligopolyenes with defined lengths. One of these [75] is relevant to the location of the 2^1^A_g_ state as a function of chain length and thus to the argument above concerning its ultimate admixture with the ground state. However, these are experimental studies and therefore the excitation energies of the 2^1^A_g_ state from the 1^1^A_g_ ground state already includes the mixing which will push the 2A_g_ state up as it is repelled by the receding 1^1^A_g_ ground state. It is, in fact, already known that in finite polyenes the two lowest ^1^A_g_ states are vibronically coupled [76,77,78,79,80]. 

## 7. Raman Vibrational Spectroscopy of Finite Polyenes

Another of these synthetic efforts concentrates on the Raman spectroscopy of finite conjugated polyenes [81]. This study is relevant to our investigation in progress of the preparation of polyacetylene *in situ* in a host–guest inclusion complex. In [81] the Raman spectra of a series of di-t-butyl polyenes with *N* = 3 to *N* = 12 C=C bonds were measured and discussed. The main observations for this series of compounds from [81] are:

- synthesis of these compounds is limited by solubility to N =12, i.e., N > 12 are insoluble;
- there are two strong Raman features near 1100 cm^−1^ and in the 1600–1500 cm^−1^ region;
- the lower C–C mode is not very sensitive to chain length;
- the higher C=C mode moves to lower frequency as the chain elongates;
- when plotted vs. 1/N, this C=C mode extrapolates to a value of ca. 1440 cm^−1^;
- the integrated intensity of the C–C mode increases relative the C=C mode as N increases.

All of the above are observed in the photochemical elimination polymerization reaction discussed below, which proceed for guest molecules in urea channel inclusion channels. In addition:

- there is a loss of mass corresponding quantitatively to the loss of iodine;
- loss of Raman intensity due to the decreasing effect of a one-electron excitation on a large chain.

## 8. In Situ Synthesis of Oriented Insulated Polyacetylene 

Polyacetylene, whatever its limitations in terms of degree of polymerization, suffers from being entirely insoluble, conformationally disordered, subject to cross linking, and having a lack of macroscopic crystallinity. All of these factors make the characterization of this material problematic. We describe here the beginnings of a method for *in situ* generation of polyacetylene in a host inclusion complex. The objective of this is to force the *trans*-polyacetylene chain to be in its fully extended all s-*trans* configuration, to prevent close proximity of neighboring polyacetylene chains and to provide exclusion of oxygen. The initial approach to this objective is the use of urea inclusion compounds containing a reactive species. Urea inclusion compounds (UICs) are self-assembling crystalline structures formed from solution with inclusion of guest hydrophobic compounds with an extended structure. The most extensive example is the series of n-alkane urea inclusion complexes. These have a macroscopic hexagonal structure with the n-alkanes being ordered in two dimensions but disordered about their axis of rotation coincident with the hexagonal c-axis. An important aspect of urea inclusion crystals for our application is that the terminal atoms of the guest species are in contact in the complex. The urea host is not stable in the absence of the guest species; the urea lattice grows around the guest species. It is known that radical polymerization can be induced in UIC’s, e.g., diene guests form very high molecular weight polymer poly(butadiene) [82,83,84]. The rigid urea tunnels allow guest rotations, translations, and lateral diffusion along the tunnel axis [82]. The guest molecules are generally more mobile than in single component molecular crystals, where reaction requires precise initial alignment. 

**Figure 9:**
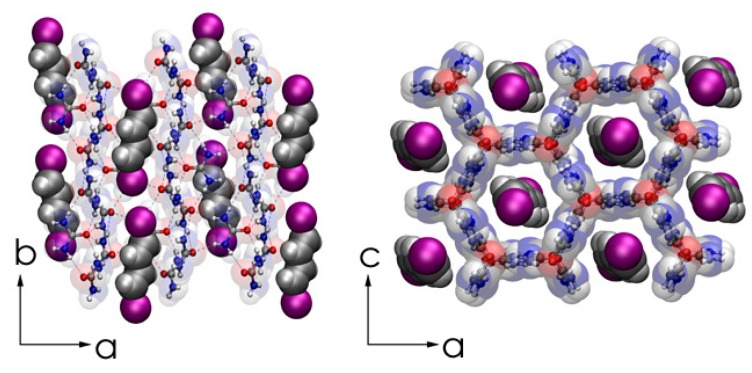
Representations of the commensurate, fully-ordered single-crystal DIBD–urea inclusion compound (UIC) complex as obtained by X-ray diffraction at 90 K viewed along the c (left panel) and b (right panel) crystal axes. Redrawn from structure Crystallographic Information File, cif of [85].

The initial focus for our experimental effort aimed at the synthesis of long chain conjugated polyenes, in particular polyacetyelene in an all*-trans* extended conformation, begun with the preparation of a crystalline urea inclusion complex with E,E–1,4–diiodo–1,3–butadiene (DIBD) [85]. In Figure 9, we present the crystal structure of DIBD–urea as obtained by X-ray diffraction at 90 K viewed perpendicular and parallel to the channel axis [85]. Like all hexagonal urea inclusions, these crystals form as parallel channels in a host lattice densely packed with guest species [82,83,84,85]. The guest monomers, DIBD, are in end-to-end contact with each other and entrapped by the hydrogen bonded urea host. The internal diameter of the channels varies periodically along the channel from 5.5 to 5.8 Å. The separation of the parallel channels is 8.2 Å. 

**Figure 10:**
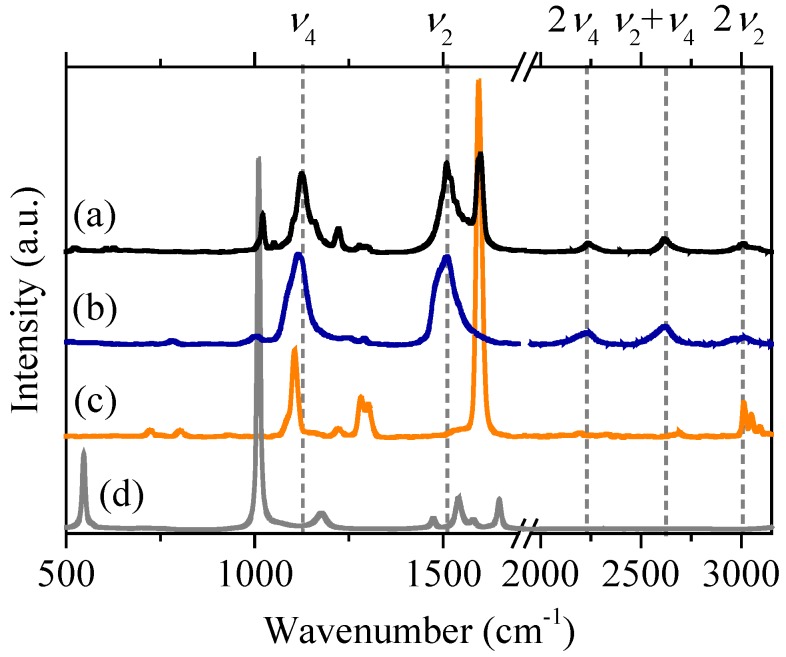
Raman spectra with 532 nm excitation of (**a**) DIBD–UIC after irradiation at 254 nm; (**b**) *trans* –(CH)_x_; (**c**) crystalline DIBD; and (**d**) tetragonal urea [86]. The ν_n_ values at the top are the mode frequencies for polyacetylene fundamental transitions and their overtones.

**Figure 11:**
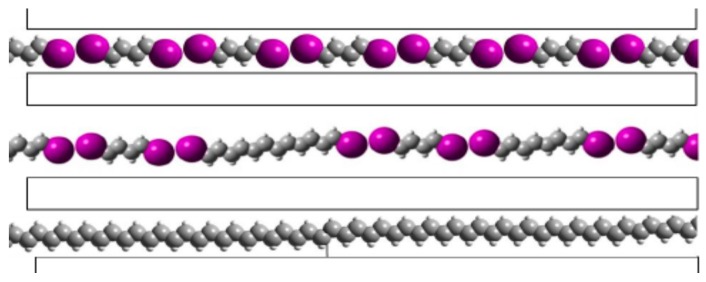
Schematic figure showing progress of the photochemical reaction from diiodobutadiene to polyacetylene with an intermediate stage showing a dimer and a trimer. The picture is to scale showing the large loss of channel filling with loss of iodine. There is a 2:1 ratio in the number of carbons in the bottom/top panels. Dimers and trimers have been shown by UV-vis of the extracted material. Longer chains have been shown by Raman.

The DIBD–urea complex used in this study is unusual in that it is a commensurate structure, in contrast to most other UICs such as those formed by n-alkanes. DIBD–urea crystals have Raman features due to DIBD at 1600 and 1250 cm^−1^. The strong tetragonal urea feature at 1010 cm^−1^ is shifted to 1022 cm^−1^, the value observed for hexagonal urea. Irradiation with ultraviolet (UV) light results in new Raman modes near 1509 and 1125 cm^−1^ [86] (Figure 10a), nearly identical to spectra of *trans*-(CH)_x_ prepared in solution [87]. The 254 nm radiation used in this experiment has very limited penetration into the DIBD–urea complex due to the high optical density. The change in composition of the urea channels is expected to be as shown in Figure 11.

**Figure 12:**
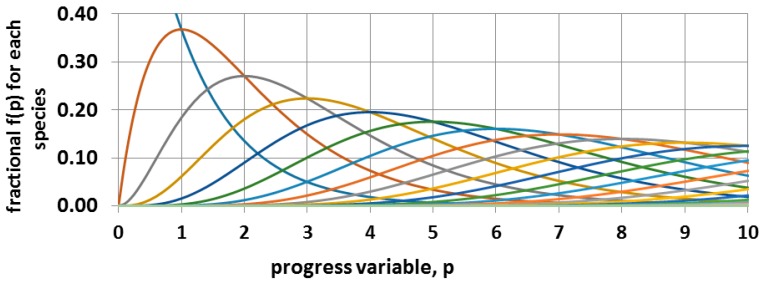
Irreversible sequential second order kinetics. The blue decreasing curve is for the monomer. The dimer peaks at p = 1 where f_2_ = f_1_, the trimer peaks at p = 2 where f_3_ = f_2_, etc. The number of carbons in the most frequent species is C_N_ = 4(p + 1). The line colors differentiate the time dependence of the sequentially larger oligomers with their increasing delay.

The overall progress of this irreversible second order sequential reaction is anticipated to be as illustrated in Figure 12. Continued progress requires that there be considerable axial diffusional motion of the chains in order to take up the space in the channel that has been vacated by the loss of iodine. This type of diffusional motion has been demonstrated with n-alkanes in UICs [88,89]. The overall progress of the reaction can be monitored with loss of mass due to release of iodine. 

## 9. Summary of Lessons from the Literature on Polyacetylene

The treatment of the vibrational level structure of polyacetylene unambiguously eliminates the possibility that bond alternation can be supported in the absence of terminal double bond end effects. Even if the barriers were much larger, the system would undergo tunneling. In reading the literature on this subject, the statement that the potential energy has a double minimum seems often to be the equivalent that the bond lengths will alternate. This is a fallacy. It is refreshing that all of the experimental methods used to establish bond alternation fail, in some cases in rather spectacular fashion. 

In the case of X-ray diffraction the nature of the samples is essentially fibers and the periodic variation of internuclear separation is a relatively fine detail to pick out reliably from such data and that further blurred by disorder. 

In the case of the NMR experiments, one wonders why the experiment was done. It could not be expected to succeed in its aim as designed, and it did not. It showed instead that polyacetylene undergoes cross-linking or some other form of conversion of double bonds to single bonds. This is also noted in the Raman studies of [35]. The better NMR experiment might be to use 50% random ^13^C labeling. 

It appears that the original interpretation of the extensive resonance Raman studies in terms of chain heterogeneity is sound with upper limits of chain length on the order of 40 double bonds.

It is our contention and that of several other workers that the Raman spectra that are observed in what is called “polyacetylene” is due to finite chains. This interpretation is consistent with observations that the most strongly enhanced feature in the Raman spectrum vary with the Raman excitation wavelength in an expected way with lower energy excitation resulting in stronger lower frequency vibrations due to preferential enhancement by longer chains. Examination of the available published data shows that there is no reliable evidence from diffraction, NMR, or IR data for bond alternation that can be demonstrated to be uncompromised by finite chains or finite conjugation segments or other ambiguities. 

Our outlook is as follows. The methodology of *in situ* synthesis of oriented insulated polyacetylene has recently taken a step forward in the preparation of urea inclusion complexes with a different guest species that has the standard hexagonal lattice and morphology. This is expected to result in much faster reaction kinetics due to slightly looser packing, ease of orientation of the crystal channel axis, and the capability of making polyacetylene chains that are the length of the crystal. This is currently ca. 1.5 cm.
